# Supplementary material for: Co-Benefits of Largescale Organic farming On huMan health (BLOOM): Protocol for a cluster-randomised controlled evaluation of the Andhra Pradesh Community-managed Natural Farming programme in India
Source: PLoS One. 2023 Mar 2;18(3):e0281677. doi: 10.1371/journal.pone.0281677 (PMC9980745; doi:10.1371/journal.pone.0281677)
Supplement: S1 Table — (DOCX) [file pone.0281677.s001.docx]

| **Supplementary Table S1.** Template for Intervention Description and Replication (TIDieR) checklist for population health and policy interventions. | |
| --- | --- |
| **TIDieR-PHP item^1^** | **BLOOM study** |
| 1 Brief name | Andhra Pradesh Community-managed Natural Farming (APCNF). |
| 2 Why | A study logic model is provided in Figure 2 explaining the mechanisms through which specific APCNF activities will have an impact on study outcomes. |
| 3 What - materials | - No hard copy informational materials will be provided to farmers or the field cadre. - No benefits (e.g., cash, seeds or other inputs) or physical resources will be provided to farmers. |
| 4 What and How | - The underpinning legislation for the intervention is an Order issued by the Government of Andhra Pradesh on 10-11-2016, entitled, “Agriculture & Cooperation Dept – Implementation of Natural Farming – Allocation of subjects to Rythu Sadhikara Samstha (Ry.S.S) – Orders – Issued.” G.O.RT No. 764. - RySS, a not-for-profit company established by the Department of Agriculture, Government of Andhra Pradesh, will fund, design, and implement the intervention. - Trainings will be delivered verbally. Field cadre will be trained virtually in groups. Farmers will be trained face-to-face both in groups and individually. |
| 5 Who provided | - RySS, a not-for-profit company established by the Department of Agriculture, Government of Andhra Pradesh, will be responsible for planning, implementation, monitoring, and enforcement of the intervention. - Specifically, internal Community Resource Persons (iCRPs) employed by RySS will be responsible for implementing the intervention. |
| 6 Where | - The intervention will be implemented at the village level. - The geographical scope of the intervention is state-wide. - The historical, socioeconomic, and political background to the intervention has been described elsewhere.^2^ |
| 7 When and how often | - The intervention will be implemented from February 2023 and will continue to be implemented for at least 2 years. - The number and duration of trainings – for both the field cadre and farmers – will be recorded prospectively and reported in a future publication. |
| - 1. Planned variation | The APCNF program is not prescriptive with regards to farmer trainings. It is designed to give the field cadre flexibility to tailor the intervention to local needs and circumstances. It is up to the iCRPs in a given village to decide which farmers in the village are interested in trying natural farming practices on their land and to respond to individual farmers’ requests for assistance. |
| 8.2 Unplanned variation | Variation in the intervention made after the intervention commences will be recorded prospectively and reported in a future publication. This could include changes to the intervention made as a result of changes in leadership, changes in policy, or new information. |
| 9.1 How well | Data collected as part of the process evaluation will be shared regularly with RySS to ensure that the intervention is delivered as intended. |
| 9.2 How well - delivery | Intervention fidelity, including variation in fidelity across villages, will be recorded prospectively and reported in a future publication. |
| ^1^Campbell M, Katikireddi SV, Hoffmann T, Armstrong R, Waters E, Craig P. TIDieR-PHP: a reporting guideline for population health and policy interventions. BMJ 2018; 361: k1079.  ^2^Veluguri D, Bump JB, Venkateshmurthy NS, Mohan S, Pulugurtha KT, Jaacks LM. Political analysis of the adoption of the Zero-Budget natural farming program in Andhra Pradesh, India. Agroecology and Sustainable Food Systems 2021; 45(6): 907-30. | |
